# Supplementary material for: Undiagnosed hypertension and associated factors among older adults in Gedeo zone, southern Ethiopia: A mixed methods approach
Source: PLoS One. 2025 May 5;20(5):e0322610. doi: 10.1371/journal.pone.0322610 (PMC12052162; doi:10.1371/journal.pone.0322610)
Supplement: S1 File — (DOCX) [file pone.0322610.s001.docx]

**Undiagnosed hypertension and associated factors among older adults in Gedeo zone, southern Ethiopia: A mixed methods approach**

Sample size calculation

We calculated the sample size for the first objective (prevalence of undiagnosed hypertension among older adults) and the second objective (factors associated with undiagnosed hypertension among older adults)

1. For the 1^st^ objective, a single population proportion formula was used

$n=\frac{\left( z_{a/2} \right)^{2} \times p(1-p)}{d^{2}}$ = $\frac{\left( 1.96 \right)^{2} \times0.5(0.5)}{{(0.05)}^{2}}$ = 384.16 ≈ 384

Where, n= sample size, p= expected proportion of undiagnosed hypertension = 0.5, we took 0.5 to obtain the maximum sample size since there has been no previously conducted research on the prevalence and associated factors of undiagnosed hypertension among older adults individuals in Ethiopia., Z= Confidence interval (95%), α= significance level (0.05), Zα /2 = the standard score for 95% confidence level is 1.96, d= Margin of error (0.05)

1. For the 2^nd^ objective, variables that had a significant association with undiagnosed hypertension among older adults were used to calculate the sample size using Epi info software.

| Variable | Outcome % in exposed | Outcome % in non-exposed | AOR | Confidence interval | power | Sample size | reference |
| --- | --- | --- | --- | --- | --- | --- | --- |
| Chronic disease (other than HTN) | 25.2 | 74.8 | 0.3 | 95% | 80% | 110 | [1] |

The exposed group for chronic disease was older adults with a chronic disease other than hypertension and non-exposed groups were older adults with no chronic disease other than hypertension.

Therefore, the sample size calculated by single population proportion is higher

- By considering the design effect (384*1.5) = 576
- By adding 10% non-response rate, 576 + 57.6 = 633.6 ≈ 633 (final sample size)

Reference

1. Zhou J, Fang S. Association between undiagnosed hypertension and health factors among middle-aged and elderly chinese population. Int J Environ Res Public Health. 2019;16(7):1214.
