# Supplementary material for: Undiagnosed hypertension and associated factors among older adults in Gedeo zone, southern Ethiopia: A mixed methods approach
Source: PLoS One. 2025 May 5;20(5):e0322610. doi: 10.1371/journal.pone.0322610 (PMC12052162; doi:10.1371/journal.pone.0322610)
Supplement: S1 Table — (DOCX) [file pone.0322610.s002.docx]

**Undiagnosed hypertension and associated factors among older adults in Gedeo zone, southern Ethiopia: A mixed methods approach**

**Supplementary Table 1: knowledge of the study participants about hypertension**

| Questions | Responses | Frequency (%) |
| --- | --- | --- |
| **Knowledge of hypertension-related risk factors** | | |
| The chance of getting HTN increased if your mother or father has HTN | No | 205 (33.7) |
|  | Yes | 201 (33.0) |
|  | I don’t know | 203 (33.3) |
| Young adults don’t get HTN | No | 176 (28.9) |
|  | Yes | 181 (29.7) |
|  | I don’t know | 252 (41.1) |
| If you are overweight, you are more likely to develop HTN | No | 129 (21.2) |
|  | Yes | 248 (40.7) |
|  | I don’t know | 232 (38.1) |
| Regular exercise helps in reducing HTN risk | No | 67 (11.0) |
|  | Yes | 337 (55.3) |
|  | I don’t know | 205 (33.7) |
| Hypertension affects men only | No | 299 (49.1) |
|  | Yes | 77 (12.6) |
|  | I don’t know | 233 (38.3) |
| Pregnancy related HTN is a situational problem and not require follow-up after delivery | No | 139 (22.8) |
|  | Yes | 73 (12.0) |
|  | I don’t know | 397 (65.2) |
| Overconsumption of salt causes HTN | No | 99 (16.3) |
|  | Yes | 297 (48.8) |
|  | I don’t know | 213 (34.9) |
| Alcohol drinking lowers the risk for HTN | No | 171 (28.1) |
|  | Yes | 192 (31.5) |
|  | I don’t know | 246 (40.4) |
| All of the following are changes that you can make in your diet to reduce risk for HTN, EXCEPT | Avoid adding table salt to food | 129 (21.2) |
|  | Eat baked food instead of fried | 113 (18.5) |
|  | Eat fast food or fried food | 73 (12.0) |
|  | I don't know | 199 (32.7) |
|  | Stop eating potato chips | 95 (15.6) |
| Cold weather increase the risk for HTN | No | 103 (16.9) |
|  | Yes | 196 (32.2) |
|  | I don’t know | 310 (50.9) |
| All of these lifestyle changes might reduce the risk for HTN, EXCEPT | Walking briskly for 30 minutes, three times a week | 186 (30.5) |
|  | Quitting cigarette smoking | 146 (24.0) |
|  | Lifting weights frequently | 126 (20.7) |
|  | I do not know | 151 (24.8) |
| **Knowledge of hypertension-related symptoms** | | |
| HTN has symptoms always | No | 217 (35.6) |
|  | Yes | 78 (12.8) |
|  | I don’t know | 314 (51.6) |
| BP is high when it is over 140/90 mmHg | No | 97 (15.9) |
|  | Yes | 307 (50.4) |
|  | I don’t know | 205 (33.7) |
| A good BP is: | 120/80 | 89 (14.6) |
|  | 140/90 | 111 (18.2) |
|  | 145/110 | 113 (18.6) |
|  | 180/100 | 86 (14.1) |
|  | I don’t know | 210 (34.5) |
| A person is diagnosed with HTN if he/she has: | A lot of headaches | 187 (30.7) |
|  | A lot of stress and tension | 94 (15.4) |
|  | Elevated blood pressure at two or more different times | 71 (11.7) |
|  | Family member(s) with high BP | 164 (26.9) |
|  | I don’t know | 93 (15.3) |
| Why is HTN called a “silent killer | HTN may have no symptoms, and can be life threatening | 34 (5.6) |
|  | The risk of dying from HTN is low | 276 (45.3) |
|  | When person does not have pain or feel ill, he/she is okay | 219 (36.0) |
|  | I don’t know | 80 (13.1) |
| **Knowledge of hypertension-related complications** | | |
| All of the following can result from high blood pressure, except | Arthritis | 94 (15.4) |
|  | Heart attack | 100 (16.4) |
|  | Kidney failure | 148 (24.3) |
|  | Stroke | 143 (23.5) |
|  | I don't know | 124 (20.4) |
| High blood pressure harms your body over time by: | Causing you to gain weight | 148 (24.3) |
|  | Causing you to have diabetes | 148 (24.3) |
|  | Damaging your blood vessels | 73 (12.0) |
|  | Making you nervous | 150 (24.6) |
|  | I don’t know | 90 (14.8) |
| HTN is not life-threatening condition | No | 96 (15.8) |
|  | Yes | 0 (0) |
|  | I don’t know | 513 (84.2) |
| **Knowledge of hypertension-related medical treatment** | | |
| There are various types of medications to treat HTN | No | 348 (57.1) |
|  | Yes | 261 (42.9) |
|  | I don’t know | 0 (0) |
| Taking blood pressure medications for long time can harm the body | No | 156 (26.1) |
|  | Yes | 122 (20.4) |
|  | I don’t know | 319 (53.4) |
| **Overall Knowledge of hypertension** | | |
| Knowledge about hypertension | Inadequate | 539 (88.5) |
|  | Adequate | 70 (11.5) |
