# Supplementary material for: Undiagnosed hypertension and associated factors among older adults in Gedeo zone, southern Ethiopia: A mixed methods approach
Source: PLoS One. 2025 May 5;20(5):e0322610. doi: 10.1371/journal.pone.0322610 (PMC12052162; doi:10.1371/journal.pone.0322610)
Supplement: Attachment — Submitted filename: PONE-D-24-27240_reviewer.pdf [file pone.0322610.s004.pdf]

PLOS ONE

The hidden burden of hypertension-undiagnosed hypertension and associated factors among elderly individuals in Gedeo zone: A mixed methods approach

--Manuscript Draft--

**Manuscript Number:**	PONE-D-24-27240
**Article Type:**	Research Article
**Full Title:**	The hidden burden of hypertension-undiagnosed hypertension and associated factors among elderly individuals in Gedeo zone: A mixed methods approach
**Short Title:**	Undiagnosed hypertension and associated factors among elderly individuals
**Corresponding Author:**	Tsion Mulat Tebeje, MPH<br>Dilla University<br>Dilla, ETHIOPIA
**Keywords:**	Undiagnosed hypertension, Elderly, Ethiopia, Gedeo zone, mixed study
**Abstract:**	Background: Hypertension has frequently remained undetected for years in settings where the healthcare system is not well developed. Undiagnosed hypertension is a significant contributing factor to the onset of cardiovascular disease, renal disease, and overall mortality and despite its increased incidence among elderly people, few studies have identified the factors contributing to it. Thus, the purpose of this research was to determine the factors related to undiagnosed hypertension among elderly individuals in the Gedeo zone, Southern Ethiopia.<br>Methods: A study design that incorporates both a community-based cross-sectional study and qualitative inquiry as a complementary approach was carried out among 609 randomly selected elderly individuals in the Gedeo zone between March 19 and May 20, 2023. A binary logistic regression model was used to determine the relationships between the outcomes and the explanatory variables. A variable was considered statistically significant if it had a p value <0.05. The qualitative data were transcribed, translated into English, and analyzed by Open Code version 4.03.<br>Results: The prevalence of undiagnosed hypertension among the elderly population was 39.24% (95% CI: 35.43%, 43.19%). The determinants that were found to have a significant relationship were living in urban areas (AOR=0.54, 95% CI: 0.34, 0.83), being able to read and write (AOR=0.21, 95% CI: 0.11, 0.38), attending primary education and above (AOR=0.53, 95% CI: 0.32, 0.87), not seeking health (AOR=2.26, 95% CI: 1.48, 3.43), being overweight or obese (AOR=4.50, 95% CI: 2.74, 7.39), having chronic diseases (AOR=1.72, 95% CI: 1.11, 2.66) and having a family history of hypertension (AOR=1.90, 95% CI: 1.13, 3.21). In-depth interviews indicated that television programs and healthcare providers are major sources of information regarding hypertension and its prevention methods.<br>Conclusion: According to this study, a significant proportion of elderly individuals in the Gedeo zone have undiagnosed hypertension. This high prevalence shows the importance of implementing a comprehensive and precise screening program for this susceptible population. Therefore, it is crucial to prioritize preventive interventions and plan appropriate programs focused on elderly people. In particular, people with chronic diseases and a family history of hypertension should be taught and encouraged to undergo timely checkups.
**Order of Authors:**	Tsion Mulat Tebeje, MPH
	Temesgen Leka Lerango, MSc
	Mihret Fikreyesus, MSc
	Daniel Sisay, MPH
**Additional Information:**	
**Question**	**Response**
**Financial Disclosure**<br><br>Enter a financial disclosure statement that	Yes

describes the sources of funding for the work included in this submission. Review the submission guidelines for detailed requirements. View published research articles from *PLOS ONE* for specific examples.<br><br>This statement is required for submission and **will appear in the published article** if the submission is accepted. Please make sure it is accurate.<br><br>**Funded studies**<br>Enter a statement with the following details:<br>• Initials of the authors who received each award<br>• Grant numbers awarded to each author<br>• The full name of each funder<br>• URL of each funder website<br>• Did the sponsors or funders play any role in the study design, data collection and analysis, decision to publish, or preparation of the manuscript?<br><br>Did you receive funding for this work?	
Please add funding details.<br>as follow-up to "**Financial Disclosure**<br><br>Enter a financial disclosure statement that describes the sources of funding for the work included in this submission. Review the submission guidelines for detailed requirements. View published research articles from *PLOS ONE* for specific examples.<br><br>This statement is required for submission and **will appear in the published article** if the submission is accepted. Please make sure it is accurate.	This research was supported by a Health Professionals Education Partnership Initiative (HEPI) grant (grant number: R25TW011214) obtained from the US National Institutes of Health, Fogarty International Center.

Powered by Editorial Manager® and ProduXion Manager® from Aries Systems Corporation

**Funded studies**<br>Enter a statement with the following details:<br>• Initials of the authors who received each award<br>• Grant numbers awarded to each author<br>• The full name of each funder<br>• URL of each funder website<br>• Did the sponsors or funders play any role in the study design, data collection and analysis, decision to publish, or preparation of the manuscript?<br><br>Did you receive funding for this work?"	
Please select the country of your main research funder (please select carefully as in some cases this is used in fee calculation).<br>as follow-up to "**Financial Disclosure**<br><br>Enter a financial disclosure statement that describes the sources of funding for the work included in this submission. Review the submission guidelines for detailed requirements. View published research articles from *PLOS ONE* for specific examples.<br><br>This statement is required for submission and **will appear in the published article** if the submission is accepted. Please make sure it is accurate.<br><br>**Funded studies**<br>Enter a statement with the following details:<br>• Initials of the authors who received each award<br>• Grant numbers awarded to each author<br>• The full name of each funder<br>• URL of each funder website<br>• Did the sponsors or funders play any role in the study design, data collection and analysis, decision to publish, or preparation of the manuscript?<br><br>Did you receive funding for this work?"	ETHIOPIA - ET
**Competing Interests**	The authors have declared that no competing interests exist.

Use the instructions below to enter a competing interest statement for this submission. On behalf of all authors, disclose any competing interests that could be perceived to bias this work—acknowledging all financial support and any other relevant financial or non-financial competing interests.<br><br>This statement is required for submission and **will appear in the published article** if the submission is accepted. Please make sure it is accurate and that any funding sources listed in your Funding Information later in the submission form are also declared in your Financial Disclosure statement.<br><br>View published research articles from *PLOS ONE* for specific examples.<br><br>**NO authors have competing interests**<br><br>Enter: *The authors have declared that no competing interests exist.*<br><br>**Authors with competing interests**<br><br>Enter competing interest details beginning with this statement:<br><br>*I have read the journal's policy and the authors of this manuscript have the following competing interests: [insert competing interests here]*<br><br>* typeset	
**Ethics Statement**<br><br>Enter an ethics statement for this submission. This statement is required if the study involved:<br><br>• Human participants<br>• Human specimens or tissue<br>• Vertebrate animals or cephalopods<br>• Vertebrate embryos or tissues	The institutional review board (IRB) of Dilla University approved the study (protocol unique number: duirb/020/23-02.

Powered by Editorial Manager® and ProduXion Manager® from Aries Systems Corporation

• Field research<br><br>Write "N/A" if the submission does not require an ethics statement.<br><br>General guidance is provided below. Consult the submission guidelines for detailed instructions. **Make sure that all information entered here is included in the Methods section of the manuscript.**	

**Format for specific study types**<br><br>**Human Subject Research (involving human participants and/or tissue)**<br>• Give the name of the institutional review board or ethics committee that approved the study<br>• Include the approval number and/or a statement indicating approval of this research<br>• Indicate the form of consent obtained (written/oral) or the reason that consent was not obtained (e.g. the data were analyzed anonymously)<br><br>**Animal Research (involving vertebrate animals, embryos or tissues)**<br>• Provide the name of the Institutional Animal Care and Use Committee (IACUC) or other relevant ethics board that reviewed the study protocol, and indicate whether they approved this research or granted a formal waiver of ethical approval<br>• Include an approval number if one was obtained<br>• If the study involved *non-human primates*, add *additional details* about animal welfare and steps taken to ameliorate suffering<br>• If anesthesia, euthanasia, or any kind of animal sacrifice is part of the study, include briefly which substances and/or methods were applied<br><br>**Field Research**<br><br>Include the following details if this study involves the collection of plant, animal, or other materials from a natural setting:<br>• Field permit number<br>• Name of the institution or relevant body that granted permission	
**Data Availability**<br><br>Authors are required to make all data underlying the findings described fully available, without restriction, and from the time of publication. PLOS allows rare exceptions to address legal and ethical concerns. See the PLOS Data Policy and FAQ for detailed information.	Yes - all data are fully available without restriction

Powered by Editorial Manager® and ProduXion Manager® from Aries Systems Corporation

A Data Availability Statement describing where the data can be found is required at submission. Your answers to this question constitute the Data Availability Statement and **will be published in the article**, if accepted.<br><br>**Important:** Stating 'data available on request from the author' is not sufficient. If your data are only available upon request, select 'No' for the first question and explain your exceptional situation in the text box.<br><br>Do the authors confirm that all data underlying the findings described in their manuscript are fully available without restriction?	
**Describe where the data may be found in full sentences. If you are copying our sample text, replace any instances of XXX with the appropriate details.**<br><br>• If the data are **held or will be held in a public repository**, include URLs, accession numbers or DOIs. If this information will only be available after acceptance, indicate this by ticking the box below. For example: *All XXX files are available from the XXX database (accession number(s) XXX, XXX.*).<br>• If the data are all contained **within the manuscript and/or Supporting Information files**, enter the following: *All relevant data are within the manuscript and its Supporting Information files.*<br>• If neither of these applies but you are able to provide **details of access elsewhere**, with or without limitations, please do so. For example:<br><br>*Data cannot be shared publicly because of [XXX]. Data are available from the XXX Institutional Data Access / Ethics Committee (contact via XXX) for researchers who meet the criteria for access to confidential data.*<br><br>*The data underlying the results presented in the study are available from (include the name of the third party*	The data are referenced in the manuscript and supplementary material.

and contact information or URL).<br>• This text is appropriate if the data are owned by a third party and authors do not have permission to share the data.<br><br>* typeset	
Additional data availability information:	

Manuscript Click here to access/download;Manuscript;Manuscript.docx

The hidden burden of hypertension-undiagnosed hypertension and associated factors among elderly individuals in Gedeo zone: A mixed methods approach

Tsion Mulat Tebeje[1*], Temesgen Leka Lerango[1], Mihret Fikreyesus[2], Daniel Sisay[1]

1. School of Public Health, College of Health Science and Medicine, Dilla University, Dilla, Ethiopia
2. Department of Nursing, College of Health Science and Medicine, Dilla University, Dilla, Ethiopia

***** Corresponding Author

Email: yemarina12@gmail.com

Abstract

Background: Hypertension has frequently remained undetected for years in settings where the healthcare system is not well developed. Undiagnosed hypertension is a significant contributing factor to the onset of cardiovascular disease, renal disease, and overall mortality and despite its increased incidence among elderly people, few studies have identified the factors contributing to it. Thus, the purpose of this research was to determine the factors related to undiagnosed hypertension among elderly individuals in the Gedeo zone, Southern Ethiopia.

Methods: A study design that incorporates both a community-based cross-sectional study and qualitative inquiry as a complementary approach was carried out among 609 randomly selected elderly individuals in the Gedeo zone between March 19 and May 20, 2023. A binary logistic regression model was used to determine the relationships between the outcomes and the explanatory variables. A variable was considered statistically significant if it had a p value <0.05. The qualitative data were transcribed, translated into English, and analyzed by Open Code version 4.03.

Results: The prevalence of undiagnosed hypertension among the elderly population was 39.24% (95% CI: 35.43%, 43.19%). The determinants that were found to have a significant relationship were living in urban areas (AOR=0.54, 95% CI: 0.34, 0.83), being able to read and write (AOR=0.21, 95% CI: 0.11, 0.38), attending primary education and above (AOR=0.53, 95% CI: 0.32, 0.87), not seeking health (AOR=2.26, 95% CI: 1.48, 3.43), being overweight or obese (AOR=4.50, 95% CI: 2.74, 7.39), having chronic diseases (AOR=1.72, 95% CI: 1.11, 2.66) and having a family history of hypertension (AOR=1.90, 95% CI: 1.13, 3.21). In-depth interviews indicated that television programs and healthcare providers are major sources of information regarding hypertension and its prevention methods.

Conclusion: According to this study, a significant proportion of elderly individuals in the Gedeo zone have undiagnosed hypertension. This high prevalence shows the importance of implementing a comprehensive and precise screening program for this susceptible population. Therefore, it is crucial to prioritize preventive interventions and plan appropriate programs focused on elderly people. In particular, people with chronic diseases and a family history of hypertension should be taught and encouraged to undergo timely checkups.

Key words: Undiagnosed hypertension, Elderly, Ethiopia, Gedeo zone, mixed study

Introduction

Non-communicable diseases (NCDs) are widely recognized as major hindrances to socioeconomic progress on a global scale. In 2019, NCDs constituted seven out of the top ten leading causes of death worldwide, marking a substantial increase compared to that in the year 2000, when only four of the top ten diseases, including hypertension and diabetes, were classified as noncommunicable [1,2]. Hypertension, also referred to as arterial hypertension, is a chronic medical condition characterized by increased blood pressure in the arteries. This condition forces the heart to exert more effort than normal to pump blood through vessels [3].

Globally, the prevalence of hypertension among adults was 31.1%, with a rate of 28.5% in high-income countries and 31.5% in low- and middle-income countries [4]. Research conducted between 2000 and 2010 revealed that the age-standardized incidence of hypertension in high-income countries decreased by 2.6%. However, in low- and middle-income countries, it increased by 7.7% [4]. Among adults with hypertension in the United States, there was no statistically significant change in hypertension control, with rates remaining at 53.9% in 2013-2014 and decreasing slightly to 48.3% in 2015-2016 [5].

Hypertension is a prominent contributor to global mortality. According to 2016 Global Health Observatory data from the WHO, high blood pressure accounts for approximately 7.5 million deaths annually, accounting for approximately 12.8% of all deaths. Furthermore, this translates to 57 million disability-adjusted life years (DALYs), representing 3.7% of the total DALYs [6]. Hypertension is a common chronic disease among elderly people, and the risk increases with age, with a prevalence of 22.4%, 54.5% and 74.5% among those aged 18–39, 40–59 and 60 and above, respectively [7].

Individuals with undiagnosed hypertension are hypertensive but have not been informed by a healthcare professional about their condition [8]. It is an issue in both developed and developing nations and contributes up to 27% of the increasing burden of cardiovascular disease [9,10]. Only 7% of those with hypertension in developing countries were conscious of and in control of their hypertension [11].

Undiagnosed hypertension significantly increases the risk of complications, including myocardial infarction, heart failure, renal failure, stroke, and premature death [12–14]. It has serious health and financial consequences; older people are disproportionately affected by high blood pressure, a recognized cardiovascular disease risk factor [15]. Early detection and diagnosis are therefore essential for managing hypertension; however, in poor nations such as Ethiopia, the majority of populations are ignorant of their condition, leading to undiagnosed, untreated, and unregulated hypertension [16]. A study in India showed that those aged above 50 years have a six-fold greater risk of undiagnosed hypertension [17]. According to a systematic review and meta-analysis conducted in Ethiopia, older individuals were at increased risk of undiagnosed hypertension [18].

The design of hypertension interventions in Ethiopia ignores the greater population of hypertensive persons who are not diagnosed, focusing primarily on those who have been diagnosed. Given that

many people with hypertension are likely to be misdiagnosed, this might have major repercussions for the nation. Despite the fact that undiagnosed hypertension is common in elderly individuals, to our knowledge, no research has been conducted in Ethiopia [19]. As a result, this study will address a gap in the literature by assessing the prevalence and related factors of undiagnosed hypertension among the elderly population. Additionally, by supplementing this study with a qualitative approach, contextual factors from the perspective of elderly people can be explored. The outcomes of this study will help institutions develop appropriate hypertension disease plans and interventions. Thus, the objective of this study was to evaluate the burden and associated factors of undiagnosed hypertension among older adults by using a mixed approach in the Gedeo zone, southern Ethiopia, in 2022.

Methods and materials

Study Area, design and Period

The study was carried out in the Gedeo Zone, which is located in the southern region of Ethiopia. 360 km from the capital of Addis Ababa. The zone encompasses 5,890.2 km2 in total. There are 5 town administrations (Dilla town, Yirgachefe, Gedeb, Wonago and Chelelektu) and 8 districts (Wonago, Repe, Yirgacheeffe, Chorso, Bule, Kochire, Dilla zuriya and Gedeb woredas) in the zone. With a crude population density of 774 people per square kilometer, the Gedeo zone has 1,226,779 people living in it overall, with a population of 107,312 elderly people. The Gedeo Zone Health Department reported that there were 250,363 households in the zone as of 2021. Between March 19 and May 20, 2023, a cross-sectional study based in the community was carried out, which was further supplemented with a qualitative study.

Population

The source population was all elderly people aged 50 years and older living in the Gedeo zone, southern Ethiopia. Elderly individuals aged 50 years and older living in the Gedeo zone who were randomly selected at the time of the study period composed the study population. The study included an elderly population who had resided in the study area for a minimum of 6 months prior to the survey, while those with severe medical conditions or mental illness were excluded.

Sample size

The single population proportion formula was used to calculate the sample size at a confidence level of 1.96 and a margin of error of 5%. We considered 50% of the population (p), as there has been no previously conducted research on the prevalence and associated factors of undiagnosed hypertension among elderly individuals in Ethiopia. Then, by taking the 1.5 design effect into account and adding a 10% nonresponse rate, the final sample size became 633. For the qualitative study, the data saturated when the number of participants reached 13.

Sampling procedure

A multistage sampling technique was utilized to select the study subjects (elderly individuals). Among the zone's five city administrations and eight districts, two districts (Dilla Zuria and Wonago woreda) and one city administration (Dilla town) were selected randomly. Then, a total of fourteen kebeles were chosen from each designated county by the lottery method to obtain 30% of the kebeles in that county. Households from each kebel were chosen proportionately according to the number of households in each chosen kebele using lists of kebeles and the number of households from zone and district officials (Figure 1). Then, systematic random sampling was conducted to select the study households. By dividing the total number of households in a particular kebele by the necessary number of participants, the selection interval (k) was

established. One house was randomly selected from each selected kebel as a starting household. The next step involved selecting households at regular intervals or every kth household as per the sampling fraction until the final household assigned to each kebele was chosen. If there was more than one eligible person aged 50 and above in the selected household, one of them was randomly selected as a study participant.

Figure 1: Schematic representation of the sampling technique used for the elderly population in the Gedeo zone, southern Ethiopia.

Study variables

The outcome variable, undiagnosed hypertension, was dichotomized into two groups: yes and no. The explanatory variables used in this study were residence, educational status, occupation, sex, marital status, household monthly income, family history of hypertension, age, khat chewing, physical exercise, cigarette smoking, alcohol consumption, comorbidities, obesity, knowledge and health-seeking behavior related to hypertension.

Measurements

Blood pressure was measured while the participants were in a sitting position using a standard mercury sphygmomanometer. The measurement was taken from the right arm, and an appropriate cuff size was used, ensuring that it covered approximately two-thirds of the upper arm. Two measurements were made 5 minutes apart. The mean systolic and diastolic blood pressures were measured in accordance with the WHO guidelines. A systolic blood pressure (SBP) measurement equal to or greater than 140 mmHg and/or a diastolic blood pressure (DBP) measurement equal to or greater than 90 mmHg were used to diagnose hypertension. Participants were categorized into three groups based on their blood pressure: (1) SBP < 120 mmHg and DBP < 80 mmHg were

considered normal blood pressure (N); (2) SBP 120-139 mmHg or DBP 80-89 mmHg was considered prehypertension but not qualifying for hypertension (HTN); and (3) hypertension: SBP/DBP >= 140/90 mmHg [20]. The participant was considered undiagnosed if the person had hypertension but did not disclose receiving a diagnosis from a medical expert.

An electronic scale or balance was used to measure weight, and a standing posture was used to assess height. Body mass index (BMI) was calculated as the weight of adults in kilograms divided by their height in meters squared. A BMI less than 18.5 kg/m2 was classified as underweight, 18.5-24.9 kg/m2 as normal, 25-29.9 kg/m2 as overweight, and 30 kg/m2 or more as obese [21].

A family history of HTN was considered if an individual's first-degree relative, such as a parent, grandparent, or sibling, had been diagnosed with hypertension and/or was receiving medication for the condition [22]. Comorbid disease is a chronic illness with a verified diagnosis of a condition other than hypertension [23].

Engaging in physical activity for 20–30 minutes, three days a week, is considered regular physical exercise [24]. The definition of current alcohol use was any amount or kind of alcohol consumed within a year before the study. An adult who had smoked cigarettes within a year before the study was referred to as a current smoker [25]. Regular khat chewing refers to chewing khat at least once a week for the previous year or longer [26].

Health-seeking behavior was defined as follows: “yes” when they claimed to visit modern health institutions (private clinics, hospitals, and health centers), “yes” when any household member was sick, or “no” otherwise [27].

Knowledge about hypertension: Knowledge of risk factors for HTN was measured by 11 items, 5 items assessed knowledge of HTN symptoms, 3 items examined knowledge of HTN

complications, and 2 items rated knowledge of HTN medical treatment. Each right response received a score of one, while wrong and I Do not Know responses received a score of zero. This created a range of 0–21 for the overall knowledge score. According to the guidelines provided by the instrument, a total knowledge percentage score of at least 70% (≥14.7 out of 21) denotes appropriate knowledge, whereas a score of less than 70% (≤14.7 out of 21) denotes inadequate knowledge. The internal consistency of the original questionnaire was 0.70 [28].

Data collection tools, procedures and quality assurance

The data collection process involved conducting face-to-face interviews using a structured questionnaire. The questionnaire was developed by drawing from various previously conducted studies and the literature. It was then adapted to suit the specific local context of the study area. The interviews aimed to gather information related to the sociodemographic status, behavioral factors, and knowledge of the study participants. It was initially written in English, and for the purpose of collecting the data, a language expert translated it into Amharic. The questionnaire was then translated back into English by a different language specialist to ensure uniformity.

We used KoboCollect to collect the data in this study. For data collection, 14 health extension workers and 3 nurses were selected for supervision. A comprehensive three-day training programme was conducted to equip data collectors and supervisors with the necessary skills and knowledge regarding the overall field survey techniques, interviewing approach, measurement, steps and process of data collection. To ensure the reliability of the data collection tools, a pretest was conducted on 5% of the total sample size in Bule Woreda 01 Kebele. This was carried out two weeks before the actual data collection period. Additionally, the principal investigator and supervisors provided daily feedback and corrections to the data collectors before they were deployed to the field the following day.

Using a semistructured interview guide, in-depth interviews (IDIs) were used to gather qualitative data. For individuals who were eligible, the purpose of the research was described. After that, the interviewers purposefully selected the participants and included them in the IDI. The population for the qualitative study was a subset of elderly individuals living in randomly selected kebeles who were not hypertensive after blood pressure measurement. Thirteen IDIs were conducted with them to understand their perceptions of the knowledge and prevention of hypertension. We obtained verbal informed consent from each subject. During each session of the in-depth interviews (IDIs), the conversations were recorded using a phone recorder. In addition, keynotes were taken to capture important points and details. The interviews took place in a private room that was specifically arranged to provide comfort and confidentiality for the participants.

Data management and analysis

The data analysis was conducted using STATA version 17, a statistical software package, to process and analyze the collected data. The data were cleaned, encoded and recoded. Appropriate descriptive statistics, such as frequency, percentage, mean, and standard deviation, were employed to examine the distribution of the data and provide a comprehensive summary. To investigate the determinants of undiagnosed hypertension, we utilized a binary logistic regression model. Initially, a bivariate binary logistic regression analysis was conducted to identify variables that were eligible for inclusion in the multivariable analysis. Variables with a p value less than 0.20 in the bivariate analysis were considered potential candidates for the multivariable binary logistic regression analysis. In the multivariable analysis, variables with a p value less than 0.05 were deemed to be statistically significant predictors. The results are presented as crude and adjusted odds ratios, accompanied by their corresponding 95% confidence intervals (CIs). To ensure that the variables were not affected by multicollinearity, we assessed the variance inflation factor (VIF) for each

variable. All variables demonstrated VIF values below 10, with a mean VIF of 1.91, indicating that multicollinearity was not a significant concern in the analysis.

For the qualitative data, all recorded interviews were transcribed verbatim to the local language Amharic. Then, the questionnaire was translated into the English language. To thoroughly understand the content, we extensively reviewed the transcripts and created concise memos and codes for each line. Subsequently, we refined and compared the newly emerged themes and subthemes. The investigators engaged in daily discussions to resolve any discrepancies, incorporated new ideas from the participants, and reached a consensus on the identified themes and subthemes. For the data analysis, we utilized open code version 4.03 software, employing a thematic content analysis approach that followed an inductive methodology.

Ethical consideration

This study was conducted following the principles outlined in the Declaration of Helsinki. The institutional review board (IRB) of Dilla University approved the study (protocol unique number: duirb/020/23-02). The survey included comprehensive information on consent, confidentiality, and the survey's objectives, which were clearly communicated on the first page. Participants were informed that their involvement was voluntary and that they had the right to withdraw their participation at any time. Prior to participation, each individual provided written informed consent. Confidentiality was strictly maintained by ensuring that the collected data did not include any personal identifiers and was solely utilized for research purposes.

Results

Characteristics of the study participants

We analyzed data on a total of 609 participants, for a 96.2% response rate. The mean age in the sample was 60.5 ±10.8 years, while the majority (63.4%) were aged between 50 and 59 years. Almost half (49.1%) of the participants were men, and the other half (50.9%) were women. The vast majority of the participants (82.6%) were married. Of the total sample, 46.6% lived in rural areas, and 37.9% had attained primary education or above. Approximately 286 (44.0%) participants were unemployed, followed by private workers (24.0%). Nearly half of the participants (49.9%) were Gedeo in ethnicity, followed by Gurage and Oromo (Table 1).

Among the respondents, 322 (52.9%) chewed khat, 160 (26.3%) smoked cigarettes, 282 (46.3%) drank alcohol, and only one-third exercised physically. Regarding body mass index, more than two-thirds of the participants had a BMI < 25 kg/m^2. More than half (55.2%) of the participants had health-seeking behavior, and 246 (40.4) had at least one chronic disease. Among the total sample, 77 (12.6%) reported a history of hypertension in their first-degree relative (Table 1).

Table 1: Characteristics of the study participants in the Gedeo zone, southern Ethiopia, 2023

Variables	Categories	Frequency	Percent (%)
Age	50-59	386	63.4
	60-69	106	17.4
	>=70	117	19.2
Sex	Female	310	50.9

	Male	299	49.1
Residence	Rural	284	46.6
	Urban	325	53.4
Marital status	Married	310	82.6
	Widowed	106	17.4
Educational status	Cannot read and write	245	40.2
	Can read and write	133	21.9
	Primary education and above	231	37.9
Occupation	Farmer	101	16.6
	Unemployed	286	44.0
	Private	146	24.0
	Others	76	12.4
Ethnicity	Gedeo	304	49.9
	Gurage	116	19.1
	Oromo	102	16.8
	Others	87	14.3
Khat chewing	Yes	322	52.9
	No	287	47.1
Cigarette smoking	Yes	160	26.3
	No	449	73.7
Alcohol drinking	Yes	282	46.3
	No	327	53.7

Physical exercise	Yes	188	30.9
	No	421	69.1
Health seeking behavior	Yes	336	55.2
	No	273	44.8
Overweight and obesity	Yes	205	33.7
	No	404	66.3
Chronic diseases	Yes	246	40.4
	No	363	59.6
Family history of HTN	Yes	77	12.6
	No	532	87.4

Participants' knowledge of hypertension

Of the participants, only 192 (31.5%) were aware that alcohol use increases the risk of hypertension. Nearly one-third (201, 33.0%) of the respondents knew that the chance of developing hypertension increased when first-degree relatives had the disease. Almost half of the participants (297, 48.8%) knew that excessive intake of salt could cause hypertension. Among the respondents, 217 (35.6) were aware that symptoms of HTN are not always present, and half (50.4%) recognized that blood pressure is deemed high if it is 140/90 mmHg or above, while only 14.6% identified that blood pressure 120/80 is considered good. Only 71 (11.7%) knew that when a person has two or more increased blood pressure readings on three distinct occasions, they may be diagnosed with HTN. The vast majority of 513 (84.2%) did not know that hypertension is a life-threatening condition, and only 73 (12%) HTN cases had detrimental effects on the body by causing damage to blood vessels. Among the participants, only 122 (20.4%) people were aware

that using antihypertensive drugs for an extended period of time damages the body, and 261 (42.9%) were aware that there are several kinds of these medications (supplementary table).

According to the instrument, a total score of hypertension knowledge below 70% (14.7) was regarded as inadequate knowledge. Overall, 539 (88.5%) of the respondents had inadequate knowledge, while the remaining 11.5% had adequate knowledge (supplementary table).

The magnitude of undiagnosed hypertension

The study participants had mean SBP and mean DBP values of 129.8 mmHg (SD = 12.0) and 84.3 mmHg (SD= 8.2), respectively. According to this study, elderly individuals had an undiagnosed HTN incidence of 39.24% (95% CI: 35.43%, 43.19%) (Figure 2).

Figure 2: Prevalence of undiagnosed hypertension among elderly individuals in the Gedeo zone, southern Ethiopia, 2023

Factors associated with undiagnosed hypertension

After adjusting for the effect of confounders, the results of multivariable binary logistic regression analysis showed that educational status, place of residence, health-seeking behavior, overweight and obesity status, family history of hypertension and the presence of at least one chronic disease were significant predictors of undiagnosed hypertension among elderly individuals. We found that elderly people who lived in urban areas (AOR=0.54, 95% CI: 0.34, 0.83) had 44% lower odds of having undiagnosed hypertension than did those who lived in rural areas. The odds of experiencing undiagnosed hypertension among elderly people who could read and write and attend primary education and above were reduced by 79% (AOR=0.21, 95% CI: 0.11, 0.38) and 47% (AOR=0.53, 95% CI: 0.32, 0.87), respectively, compared to elderly people who could not read and write. In contrast to elderly individuals who exhibited health-seeking behavior, those who did not have HSB

had 2.26-fold (AOR=2.26, 95% CI: 1.48, 3.43) greater odds of undiagnosed hypertension. Compared to elderly individuals who were neither overweight nor obese, those who were overweight or obese were 4.5 times (AOR=4.50, 95% CI: 2.74, 7.39) more likely to have undiagnosed hypertension. Additionally, the likelihood of undiagnosed hypertension was 72% and 90% greater for elderly individuals with chronic diseases (AOR=1.72, 95% CI: 1.11, 2.66) and a family history of hypertension (AOR=1.90, 95% CI: 1.13, 3.21), respectively, than for their counterparts (Table 2).

Table 2: Multivariable binary logistic regression analysis of factors associated with undiagnosed hypertension among elderly individuals in the Gedeo zone, 2023

Variables	Category	Undiagnosed hypertension Number (%)	COR (95%CI)	AOR (95%CI)
Sex	Female	130 (54.4)	1	1
	Male	109 (45.6)	0.74 (0.57, 1.10)	1.53 (0.99, 2.34)
Residence	Rural	119 (49.8)	1	1
	Urban	120 (50.2)	0.81 (0.59, 1.12)	**0.54 (0.34, 0.83)****
Educational status	cannot read and write	112 (46.9)	1	1
	can read n write	39 (16.3)	0.49 (0.31, 0.77)	**0.21 (0.11, 0.38)*****
	primary education and above	88 (36.8)	0.73 (0.51, 1.05)	**0.53 (0.32, 0.87)***
Occupation	Farmer	33 (13.8)	1	1

	Unemployed	139 (58.2)	1.95 (1.21, 3.14)	1.57 (0.92, 2.67)
	Private	39 (16.3)	0.75 (0.43, 1.31)	0.57 (0.31, 1.06)
	Others	28 (11.7)	1.20 (0.64, 2.24)	1.38 (0.66, 2.87)
Health seeking behavior	Yes	109 (45.6)	1	1
	No	130 (54.4)	1.89 (1.36, 2.63)	**2.26 (1.48, 3.43)*** **
Overweight and Obesity	No	53 (22.2)	1	1
	Yes	186 (77.8)	2.45 (1.69, 3.54)	**4.50 (2.74, 7.39)*** **
Chronic disease	No	112 (46.9)	1	1
	Yes	127 (53.1)	2.39 (1.71, 3.34)	**1.72 (1.11, 2.66)***
Family history	No	193 (80.8)	1	1
	Yes	46 (19.3)	2.08 (1.31, 3.31)	**1.90 (1.13, 3.21)***

*** < 0.001, ** < 0.01 and * <0.05

A key informant interview was conducted, and the data were saturated when the number of participants reached 13. Eight of them were female, while the rest were male. After reading the transcripts, two themes were developed that had different subthemes.

Almost all of the participants were aware that hypertension occurred when blood vessel pressure was higher than usual. However, many are unaware of what a normal blood pressure level is. Only a few participants stated that hypertension occurs when the pressure in the blood vessel is more than 120/80. The majority of them learned about the definition of hypertension from watching health-related TV shows. Others heard about it from other friends, relatives and coworkers who had the disease and from medical professionals. This is how one participant put it.

"Regarding my blood pressure situation…. I know the normal reading for blood pressure is 120/80, and it should not be more than that... If it is greater than this, I understand it is not a good thing. In addition, I always measure my blood pressure…I heard this from a TV show at one time." (Participant 1, Male age 58)

The majority of participants listed headache and dizziness as common symptoms of hypertension. In addition, they were only able to state the aforementioned symptoms. Fainting and blurred vision were mentioned as signs of high blood pressure by only two of the participants. The majority of participants stated that they would be anxious to learn that they had the illness since it could lead to serious complications and require them to modify their diet and activities. They become increasingly anxious about being diagnosed with this disease, as they see the illness as causing more health issues and even death in other people. A few mentioned that it might happen to them, and since it can happen to anyone, it is nothing to be alarmed about; all they need to do is take their medication and adjust their way of living. One of the participants expressed that she would be very stressed if she were to be diagnosed with hypertension as follows:

"I lost my father ten years ago due to hypertension. I truly hate that disease…he was just fine the whole day and he got angry at night because of something and he fainted…after that he was admitted to hospital for 10 days...the doctors said he had a stroke….he could not get of the hospital alive…so I don't want to be diagnosed with the disease." (Participant 10, female 52 years)

Another participant described it as follows: *"Hypertension is a silent killer. I heard from my friend, families… I don't what it may bring on me. So I take care of myself as much as possible." (Participant 5, male 70 years)*

The majority of participants indicated that by engaging in various preventive activities, they could avoid hypertension. Most of them stated that as they get older, all they need to do is reduce their salt intake, stress, and exercise. Few participants claimed that cutting back on alcohol, coffee, and fatty meats would help them avoid the illness. One participant said that to avoid the disease, he must maintain his weight within a normal range for his height and age. One of the interviewees put it this way:

"I don't know it if it is scientific or not. However, I know there are three things that are said to be deadly and lead to increased blood pressure… the three white…Like salt, sugar and fatty meat… I will control myself from these things" (Participant 6, male 65 years).

The participants were asked what they would do if they were to be diagnosed with hypertension. They all said they needed to exercise, reduce salt and follow the advice of health care providers. Half of the participants added that patients must adhere to health care providers' orders and take their medication as prescribed strictly. Only two participants stated that they needed to reduce their alcohol and coffee intake, stop smoking cigarettes, and start checking their blood pressure more frequently. One participant expressed it as follows:

"It is like what I said before. By doing physical exercise and modifying one's feeding style…avoiding different stressful things...taking things easily…people can manage high blood pressure" (participant 12, male 70 years old).

Discussion

Older adults are at increased risk of hypertension. As age increases, the vascular system undergoes changes that result in stiffening of the arteries, leading to a rise in blood pressure [29]. The purpose of this study was to determine the prevalence of and factors associated with undiagnosed

hypertension among elderly people by conducting qualitative and quantitative cross-sectional studies. According to the current study, 39.24% (95% CI: 35.43%, 43.19%) of the elderly population had undiagnosed hypertension. In other words, more than one-third of the individuals included in the study did not know about their disease. This finding is greater than those of studies performed among elderly people in Iran [30] and China [31]. The disparity could be attributed to socioeconomic differences between countries. Compared with those countries, Ethiopia has lower education, healthcare system, infrastructure and access to medical services, which increases the magnitude of undiagnosed hypertension [32]. However, this value is lower than that reported in a study from Bangladesh [33]. This discrepancy could be explained by the difference in the scope of the study and population, as their participants were aged 35+ years.

The results of the multivariable binary logistic regression model revealed that elderly individuals who lived in urban areas had a lower likelihood of having undiagnosed hypertension than did those who lived in rural areas. This finding aligns with previous studies performed in India [34], China [31] and Malaysia [35]. This is because urban regions have more access to healthcare facilities than do rural regions. Additionally, the inaccessibility of healthcare facilities as a result of poor transport and communication as well as the poor use of health care services in rural regions due to reliance on traditional medicines contributes to undiagnosed hypertension [36,37].

Consistent with previous studies [35,38,39], elderly people who can read and write and/or attend primary education have greater chances of having undiagnosed hypertension than elderly people who are unable to read and write. This shows that a low educational level is associated with a lack of awareness and limited understanding of health-related information. It was also found that compared to elderly individuals who exhibit health-seeking behavior, those with no health-seeking behavior are at high risk of having their hypertension undiagnosed. This is corroborated by studies

from Ethiopia [12,40]. The possible reason is that those who have low healthcare seeking behavior are less likely to visit health institutions to undergo hypertension screening.

Our findings support those of studies conducted in Ethiopia [40–43], which reported higher odds of undiagnosed hypertension among those who were overweight or obese. Excess body weight is closely linked to an increased risk of cardiovascular morbidity and an elevated cardiovascular risk. There is a well-established association between being overweight and the activation of the sympathetic nervous system and the renin-angiotensin system, leading to the development of hypertension. Neuroendocrine pathways are the main way that obesity and hypertension are related, and recent research has suggested that factors originating from adipose tissue play a significant role in this relationship [44,45].

Chronic disease was also found to have a significant influence on undiagnosed hypertension. In this regard, elderly individuals with any chronic disease had increased odds of undiagnosed hypertension. This finding was consistent with the results of previous studies [18,22,40,42]. For instance, among those with diabetes mellitus, elevated blood glucose levels can indeed cause extensive damage to blood vessels, increase fluid volume, and promote insulin resistance, all of which are key mechanisms contributing to the increased risk of hypertension [46].

Furthermore, the likelihood of undiagnosed hypertension was greater among elderly individuals with a family history of hypertension. This was in agreement with studies from Ethiopia [18,22,41], and a possible explanation might be that the greater chance of undiagnosed hypertension among those who have a family history of HTN may be linked to genetic factors that increase the risk of developing high blood pressure. The underlying genetic mechanisms are likely to involve variations in multiple genes that impact the regulation of blood pressure and cardiovascular system function [47].

In the qualitative study, none of the participants knew the scientific and correct definition of high blood pressure. They simply understand that it is an increase in blood pressure. Few participants stated that hypertension occurs when their blood pressure is greater than 120/80. They learned this from television shows, health care providers, and others around them, which is similar to the findings of a study performed in Uganda [48]. However, in Malaysia, most of the participants acquired their knowledge about hypertension from the internet [49]. All participants identified headaches and dizziness as the most common symptoms of hypertension, with only a few participants mentioning additional symptoms such as blurred vision and fainting, similar to the findings of a study performed in Malaysia [49]. However, none of them were aware of additional symptoms such as chest pain, difficulty breathing, vomiting, nausea, or other symptoms. The majority of participants are afraid of acquiring this disease because it causes additional complications, sudden death and lifestyle changes, similar to a study performed in Uganda [48].

The majority of participants were aware of disease prevention measures. They stated that they should exercise more, consume less salt, and avoid stress. Others stated that they needed to reduce their consumption of alcohol and cigarettes. One participant stated that maintaining a normal weight-to-height ratio is one way to prevent the disease. This finding is in line with research performed in Aksum, Ethiopia [50]. Likewise, the majority of participants stated that if they were diagnosed with hypertension, they would simply need to adjust their lifestyle based on the advice of their doctors and strictly adhere to their medication. Others stated that physical activity will help manage the disease, which is comparable to the findings of a study carried out in Uganda [48].

Strengths and limitations

The study was not free of limitations. It is difficult to ascertain the cause-and-effect relationship because the study was cross-sectional. Another drawback might be participant reporting bias. The

community-based nature of the study and the large sample size used were strengths. Standard guidelines were used to diagnose hypertension and other variables.

Conclusion

This study revealed the high prevalence of undiagnosed hypertension among elderly individuals in the Gedeo zone of Ethiopia and showed the importance of implementing a comprehensive and precise screening program for this susceptible population. Place of residence, educational status, health-seeking behavior, overweight and obesity status, family history of hypertension, and presence of at least one chronic disease were significantly associated with undiagnosed hypertension among elderly people. Thus, by considering the increasing number of elderly people and their illiteracy in low-income countries, including Ethiopia, prioritizing preventative measures and creating suitable programs focused on elderly people are important. In particular, people with chronic diseases and a family history of hypertension should be taught and encouraged to undergo timely checkups. Public awareness campaigns on hypertension should also be developed and funded through media channels and platforms, including radio and television programs, to address the elderly population. Furthermore, making choices for a healthy lifestyle should also be carefully considered, especially in the last stages of life.

List of abbreviations

AOR: adjusted odds ratio

BMI: Body mass index

CI: Confidence interval

DALY: Disability Adjusted Life Year

DBP: diastolic blood pressure

HEPIs: Health Professionals Education Partnership Initiatives

HTN: systolic blood pressure

IRB: Institutional Review Board

NCD: Non-Communicable Disease

SBP: systolic blood pressure

VIF: Variance Inflation Factor

Declarations

Consent for publication

Not applicable

Availability of data and materials

The data are referenced in the article and supplementary material.

Competing interests

The authors declare they have no competing interests.

Funding

This research was supported by a Health Professionals Education Partnership Initiative (HEPI) grant (grant number: R25TW011214) obtained from the US National Institutes of Health, Fogarty International Center.

Acknowledgments

We would like to acknowledge the HEPI initiative for providing funds to conduct this study.

Author Contributions

Tsion Mulat Tebeje & Daniel Sisay: Conceived the study, analyzed the data, wrote the original draft, and revised and edited the paper.

Mihret Fikreyesus & Temesgen Leka Lerango: Analyzed the interpreted data and revised and edited the paper.

References

1. Bollyky TJ, Templin T, Cohen M, Dieleman JL. Lower-income countries that face the most rapid shift in noncommunicable disease burden are also the least prepared. Health Aff. 2017;36(11):1866–75.

2. Nibret E, Worku G, Zewdie A. The Prevalence of Chronic Kidney Disease and Associated Factors among Type-2 Diabetic Patients Admitted to Dilla University Referral Hospital Medical …. Morb Mortal [Internet]. 2020;10:11. Available from: https://www.sciresliterature.org/Immunology/SJII-ID22.pdf

3. Awoke A, Awoke T, Alemu S, Megabiaw B. Prevalence and associated factors of hypertension among adults in Gondar, Northwest Ethiopia: A community based cross-sectional study. BMC Cardiovasc Disord. 2012;12(1):1.

4. Kearney PM, Whelton M, Reynolds K, Whelton PK HJ. Kearney PM, Whelton M, Reynolds K, Whelton PK, He J (January 2004). “Worldwide prevalence of hypertension: a systematic review”. J. Hypertension. 22 (1): 11–9.

5. Fryar CD, Ostchega Y, Hales CM, Zhang G, Kruszon-Moran D. Hypertension prevalence and control among adults: United States, 2015-2016. 2017;

6. Organization WH. Ghana country assessment report on aging and health. Geneva, Switzerland: Author. 2014.

7. Ostchega Y, Fryar CD, Nwankwo T, Nguyen DT. Hypertension Prevalence Among Adults Aged 18 and Over: United States, 2017-2018. NCHS Data Brief. 2020 Apr;(364):1–8.

8. Choo EH, Ihm SH, Lim S, Chang K, Seung KB. A simple screening score for undiagnosed hypertension. Vol. 172, International journal of cardiology. Netherlands; 2014. p. e465-7.

9. Dejenie M, Kerie S, Reba K. Undiagnosed hypertension and associated factors among bank workers in Bahir Dar City, Northwest, Ethiopia, 2020. A cross-sectional study. PLoS One. 2021;16(5):e0252298.

10. Ataklte F, Erqou S, Kaptoge S, Taye B, Echouffo-Tcheugui JB, Kengne AP. Burden of undiagnosed hypertension in subsaharan africa: A systematic review and meta-analysis. Hypertension. 2015;65(2):291–8.

11. Thapliyal V, Singh K, Joshi A. Prevalence and associated factors of hypertension among adults in rural Uttarakhand: A community based cross sectional study. Curr Res Nutr Food Sci. 2018;6(2):481–90.

12. Haligamo D, Ayalew A, Genemo H, Yiriga N. Undiagnosed Hypertension and Its Associated Factors Among Adult People Living in Southern Ethiopia: Discovery From Gunchire Woreda of Gurage Zone. J Manag Strateg. 2021;12(4):37.

13. Misganaw A, Melaku YA, Tessema GA, Deribew A, Deribe K, Abera SF, et al. National disability-adjusted life years (DALYs) for 257 diseases and injuries in Ethiopia, 1990-2015: Findings from the global burden of disease study 2015. Popul Health Metr. 2017;15(1):1–16.

14. Tesfaye F, Byass P, Wall S. Populationbased prevalence of high blood pressure among adults in Addis Ababa: Uncovering a silent epidemic. BMC Cardiovasc Disord. 2009;9(1):1–10.

15. Mokona H, Yohannes K, Ayano G. Youth unemployment and mental health: Prevalence and associated factors of depression among unemployed young adults in Gedeo zone, Southern Ethiopia. Int J Ment Health Syst. 2020;14(1):1–11.

16. Mogas SB, Tesfaye T, Zewde B, Tesfaye Y, Kebede A, Tadesse M, et al. Burden of undiagnosed hypertension among adults in urban communities of southwest Ethiopia. Integr Blood Press Control. 2021;14:69–76.

17. Appadurai PD, Rajanayagam ARN, Asharaf RM, Govindan PP. Undiagnosed hypertension and its correlates among adults attending urban and rural health training centers in a South Indian district. J Educ Health Promot. 2023;12:162.

18. Ambaw Kassie G, Alemu A, Yosef Gebrekidan A, Asmare Adella G, Eshetu K, Wolie Asres A, et al. Undiagnosed hypertension and associated factors among adults in ethiopia: a systematic review and meta-analysis. BMC Cardiovasc Disord. 2023 May;23(1):278.

19. Raz N, Rodrigue KM, Acker JD. Hypertension and the Brain: Vulnerability of the Prefrontal Regions and Executive Functions. Behav Neurosci. 2003;117(6):1169–80.

20. Chobanian A V, Bakris GL, Black HR, Cushman WC, Green LA, Izzo JLJ, et al. Seventh report of the Joint National Committee on Prevention, Detection, Evaluation, and Treatment of High Blood Pressure. Hypertens (Dallas, Tex 1979). 2003 Dec;42(6):1206–52.

21. Tesfaye Yunka T, Berhanu Mogas S, Zawdie B, Tamiru D, Tesfaye Y, Kebede A, et al. The Hidden Burden of Diabetes Mellitus in an Urban Community of Southwest Ethiopia. Diabetes Metab Syndr Obes. 2020;13:2925–33.

22. Wachamo D, Geleta D, Woldesemayat EM. Undiagnosed Hypertension and Associated Factors Among Adults in Hawela Tula Sub-City, Hawassa, Southern Ethiopia: A Community-Based Cross-Sectional Study. Risk Manag Healthc Policy. 2020;13:2169–77.

23. Liu H, Chen S, Liu M, Nie H, Lu H. Comorbid Chronic Diseases are Strongly Correlated with Disease Severity among COVID-19 Patients: A Systematic Review and Meta-Analysis. Aging Dis. 2020 May;11(3):668–78.

24. Tefera BZ, Zeleke H, Abate A, Abebe H, Mekonnen Z, Sewale Y. Magnitude and associated factors of low back pain among nurses working at intensive care unit of public hospitals in Amhara region, Ethiopia. PLoS One. 2021;16(12):e0260361.

25. Federal Ministry of Health of Ethiopia. Guidelines on clinical and programmatic management of major noncommunicable diseases. ResearchGate. 2016;(August):220.

26. Astatkie A, Demissie M, Berhane Y, Worku A. Prevalence of and factors associated with regular khat chewing among university students in Ethiopia. Subst Abuse Rehabil. 2015;6:41–50.

27. Begashaw B, Tessema F, Gesesew HA. Health care seeking behavior in Southwest Ethiopia. PLoS One. 2016;11(9):e0161014.

28. Han HR, Chan K, Song H, Nguyen T, Lee JE, Kim MT. Development and evaluation of a hypertension knowledge test for Korean hypertensive patients. J Clin Hypertens. 2011;13(10):750–7.

29. Sri Hari TY, Sree Sudha TY, Varghese AM, Krishna Sasanka KSBS, Thangaraju P. A study of risk factors and complications in elderly hypertensive subjects. J Fam Med Prim Care [Internet]. 2021;10(6). Available from: https://journals.lww.com/jfmpc/fulltext/2021/10060/a_study_of_risk_factors_and_complications_in.18.aspx

30. Seyed Reza SR, Bayani M, Zabihi A, Shakerian M, Habibian T, Bijani A. Undiagnosed hypertension and diabetes among the elderly in Amirkola, North of Iran. Casp J Intern Med. 2019;10(3):303–8.

31. Zhou J, Fang S. Association between undiagnosed hypertension and health factors among middle-aged and elderly chinese population. Int J Environ Res Public Health. 2019;16(7):1214.

32. Eregata GT, Hailu A, Memirie ST, Norheim OF. Measuring progress toward universal health coverage: national and subnational analysis in Ethiopia. BMJ Glob Heal [Internet]. 2019 Nov 1;4(6):e001843. Available from: http://gh.bmj.com/content/4/6/e001843.abstract

33. Ahmed S, Tariqujjaman M, Rahman MA, Hasan MZ, Hasan MM. Inequalities in the prevalence of undiagnosed hypertension among Bangladeshi adults: evidence from a nationwide survey. Int J Equity Health [Internet]. 2019;18(1):33. Available from: https://doi.org/10.1186/s12939-019-0930-5

34. Boro B, Banerjee S. Decomposing the rural–urban gap in the prevalence of undiagnosed, untreated and undertreated hypertension among older adults in India. BMC Public Health

[Internet]. 2022;22(1):1310. Available from: https://doi.org/10.1186/s12889-022-13664-1

35. Lim OW, Yong CC. The Risk Factors for Undiagnosed and Known Hypertension among Malaysians. Malays J Med Sci. 2019 Sep;26(5):98–112.

36. Dey S, Nambiar D, Josyula L, Sheikh K, Reddy KS. Health of the elderly in India: Challenges of access and affordability. In: Aging in Asia: Findings from new and emerging data initiatives. 2012.

37. Boro B, Saikia N. A qualitative study of the barriers to utilizing healthcare services among the tribal population in Assam. PLoS One [Internet]. 2020 Oct 8;15(10):e0240096. Available from: https://doi.org/10.1371/journal.pone.0240096

38. Zacher M. Educational Disparities in Hypertension Prevalence and Blood Pressure Percentiles in the Health and Retirement Study. J Gerontol B Psychol Sci Soc Sci. 2023 Aug;78(9):1535–44.

39. Bushara SO, Noor SK, Elmadhoun WM, Sulaiman AA, Ahmed MH. Undiagnosed hypertension in a rural community in Sudan and association with some features of the metabolic syndrome: how serious is the situation? Ren Fail [Internet]. 2015 Jul 3;37(6):1022–6. Available from: https://doi.org/10.3109/0886022X.2015.1052951

40. Regea F, Birhanu A, Shibiru A. Distribution of risks and prevalence of unscreened hypertension among adults living in rural of Dano District, West Shewa, Oromia, Ethiopia, 2020: Community based cross-sectional study. Clin Epidemiol Glob Heal [Internet]. 2022;13:100908. Available from: https://www.sciencedirect.com/science/article/pii/S2213398421002165

41. Essa E, Shitie D, Yirsaw MT, Wale MZ. Undiagnosed hypertension and associated factors among adults in Debre Markos town, North–West Ethiopia: A community-based cross-sectional study. SAGE open Med. 2022;10:20503121221094224.

42. Ayalew TL, Wale BG, Zewudie BT. Burden of undiagnosed hypertension and associated factors among adult populations in Wolaita Sodo Town, Wolaita Zone, Southern Ethiopia. BMC Cardiovasc Disord. 2022 Jun;22(1):293.

43. Elias S, Dadi TK. Prevalence of Undiagnosed Hypertension and Associated Factors among Adults in Mizan-Aman Town, Bench Sheko Zone, Southwest Ethiopia: A Community-Based Cross-Sectional Study. Adv Med. 2023;2023:2746284.

44. Limpijankit T, Vathesatogkit P, Matchariyakul D, Wiriyatanakorn S, Siriyotha S, Thakkinstian A, et al. Causal relationship of excess body weight on cardiovascular events through risk factors. Sci Rep [Internet]. 2022;12(1):5269. Available from: https://doi.org/10.1038/s41598-022-08812-x

45. Ashraf MJ, Baweja P. Obesity: the “enormous” problem in cardiovascular diseases. Mo Med. 2013;110(6):499–504.

46. Cheung BMY, Li C. Diabetes and hypertension: is there a common metabolic pathway? Curr Atheroscler Rep. 2012 Apr;14(2):160–6.

47. Liu M, He Y, Jiang B, Wang J, Wu L, Wang Y, et al. Association Between Family

History and Hypertension Among Chinese Elderly. Medicine (Baltimore) [Internet]. 2015;94(48). Available from: https://journals.lww.com/md-journal/fulltext/2015/12010/association_between_family_history_and.27.aspx

48. Lynch HM, Green AS, Clarke Nanyonga R, Gadikota-Klumpers DD, Squires A, Schwartz JI, et al. Exploring patient experiences with and attitudes toward hypertension at a private hospital in Uganda: a qualitative study. Int J Equity Health [Internet]. 2019;18(1):206. Available from: https://doi.org/10.1186/s12939-019-1109-9

49. Tan CS, Hassali MA, Neoh CF, Saleem F. A qualitative exploration of hypertensive patients' perception toward quality use of medication and hypertension management at the community level. Pharm Pract (Granada). 2017;15(4):1074.

50. Gebrihet TA, Mesgna KH, Gebregiorgis YS, Kahsay AB, Weldehaweria NB, Weldu MG. Awareness, treatment, and control of hypertension is low among adults in Aksum town, northern Ethiopia: A sequential quantitative–qualitative study. PLoS One. 2017;12(5):e0176904.

Figure

Click here to access/download;Figure;Figure 1.tif

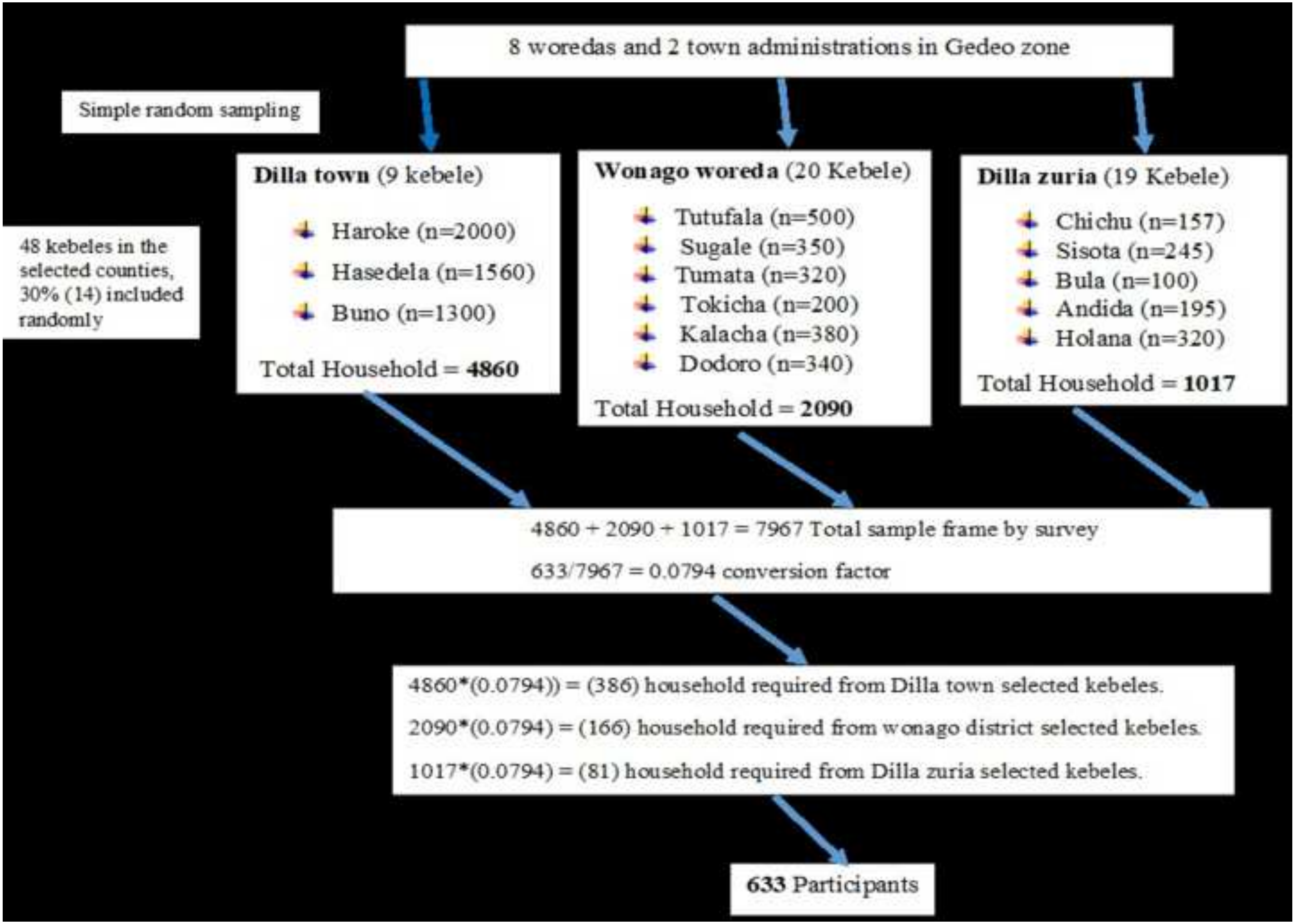

Figure

Click here to access/download;Figure;Figure 2.tif

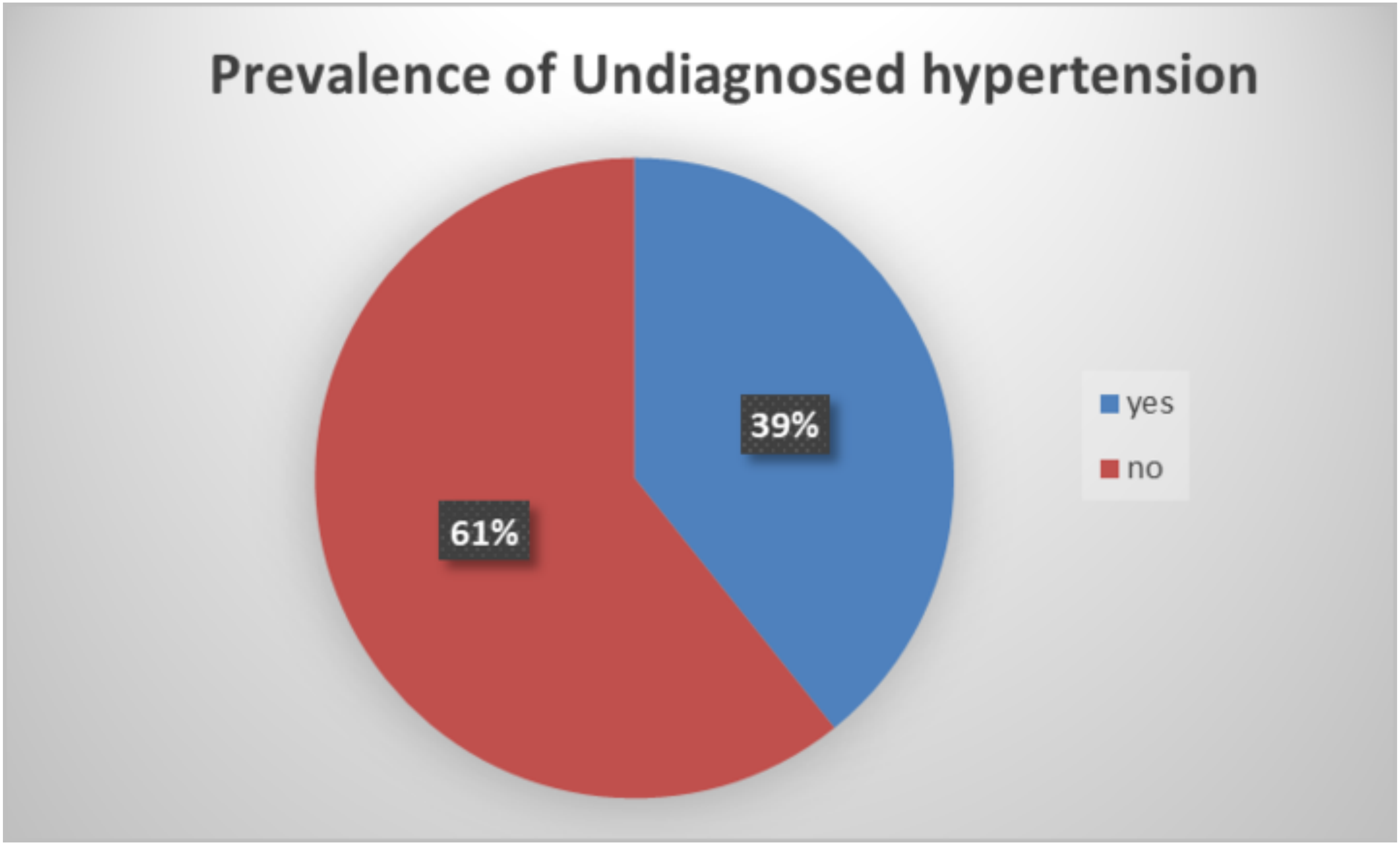

Click here to access/download
Supporting Information
supplementary table.docx
