# Supplementary material for: Undiagnosed hypertension and associated factors among older adults in Gedeo zone, southern Ethiopia: A mixed methods approach
Source: PLoS One. 2025 May 5;20(5):e0322610. doi: 10.1371/journal.pone.0322610 (PMC12052162; doi:10.1371/journal.pone.0322610)
Supplement: Attachment — Submitted filename: Comment.pdf [file pone.0322610.s005.pdf]

Comment:

The hidden burden of hypertension-undiagnosed hypertension and associated factors among elderly individuals in Gedeo zone: A mixed methods approach

Manuscript Draft

1. From the title line 1, there is a reputation for “hypertension-undiagnosed hypertension”, please rewrite again to get more credit or approval?
2. The abstract section line [13-14] points out that the only reason for the failure of the health system is that the cause of hypertension is not detected, please see other published documents (https://doi.org/10.1186/s12872-022-02733-3)
3. Why are you selecting the elderly, please give real evidence of the selection of the elderly compared to other elderly?
4. Why choose only the Gedeo zone, southern Ethiopia, why not include other zones under southern Ethiopia?
5. In the section 19 of the method carried out among 609 randomly selected elderly people in the Gedeo area between 19 March and 20 May 2023, is this sample sufficient? Please analyze to add additional samples to get a more accurate result?
6. From line 27 of page 2 “The determinants that had a significant relationship were living in urban areas,” what about rural areas under the Gedeo region of southern Ethiopia?, because your study focused on the Gedeo region of southern Ethiopia, so you should also include rural populations.
7. In the result part line 26 pages 2 the last sentence is [33-34] is not appropriate position is better to write in the conclusion part.
8. From page 3 of the introduction paragraph 46, first sentences, "Non-communicable diseases (NCDs) are widely recognized as major obstacles to socio-economic progress worldwide," please give evidence or references to show its effect.
9. From the introduction part line [50-56] is about adults, but your investigation of older people, so the two ideas contradict, so please try to write a detailed introduction about older people? I do not think it is important to consider adult causes.
10. From line [81-82], “Despite the fact that undiagnosed hypertension is common in older people, to our knowledge, no research has been conducted in Ethiopia [19]’’, from this part there is no need for reference, because the sentence indicates your idea.

11. From line 6 [110-11], the phrase says: “As there has been no previous research on the prevalence and associated factors of undiagnosed hypertension among elderly people in Ethiopia.”, your study area focused only on a restricted Gedeo area, southern Ethiopia, but there are so many studies done in Ethiopia, Africa and other countries, so how unique is your research from other research?
12. From line 6[117-119] of page 6 the sentence says: "Then a total of 14 kebeles were chosen from each designated county by the lottery method to obtain 30% of the kebeles in that county", is that a standardized accepted method for the use of the lottery method?
13. On page 11 of line 216, the phrase states "For qualitative data, all interviews recorded have been verbatim translated into the local Amharic language", but your research area Gedeo zone, southern Ethiopia, why choose Amharic language?, because Gedeo zone, southern Ethiopia has its own language.
14. From page 22 in line 414, the sentence says, "Another disadvantage may be the participant's reporting of prejudices," how do you know it and how to solve it? Because reporting biases occur, there is a question about data quality.
15. From the reference, please use the most recently published document not the oldest published document? For example, in 2012 more than a decade, so it is not a relevant reference.
