# Supplementary material for: Undiagnosed hypertension and associated factors among older adults in Gedeo zone, southern Ethiopia: A mixed methods approach
Source: PLoS One. 2025 May 5;20(5):e0322610. doi: 10.1371/journal.pone.0322610 (PMC12052162; doi:10.1371/journal.pone.0322610)
Supplement: Attachment — Submitted filename: Response to reviewers.docx [file pone.0322610.s006.docx]

**Subject: Responses to comments**

Dear Editor,

Thank you for taking the time to consider our manuscript titled **“**Undiagnosed hypertension and associated factors among older adults in Gedeo zone, southern Ethiopia: A mixed methods approach**”** for the Plos One Journal original research article. We appreciate the time and effort you and the reviewers have dedicated to providing valuable feedback on our manuscript.

We have considered the comments and concerns and made every effort to address them. We agree with all the comments and have incorporated the corresponding revisions into the revised manuscript. All revised texts are track-changed to point out the changes we made. We believe that our manuscript has been significantly improved as a result of these revisions.

We would like to thank you once again for your consideration of our work and for inviting us to submit the revised manuscript. We look forward to hearing from you. Our detailed, point-by-point responses to the comments are given below.

Best regards,

Tsion Mulat Tebeje

School of Public Health, Dilla University, Dilla, Ethiopia.

Email: [yemarina12@gmail.com](mailto:yemarina12@gmail.com) / [Tsionmulat@du.edu.et](mailto:Tsionmulat@du.edu.et) (corresponding author)

**Response to journal’s requirements**

1. Please ensure that your manuscript meets PLOS ONE's style requirements, including those for file naming.

**Response**: We prepared the manuscript according to the journal's requirements and double-checked its compliance before submitting our revised manuscript.

2. Thank you for stating the following financial disclosure:

“This research was supported by a Health Professionals Education Partnership Initiative (HEPI) grant (grant number: R25TW011214) obtained from the US National Institutes of Health, Fogarty International Center.”

Please state what role the funders took in the study. If the funders had no role, please state: "The funders had no role in study design, data collection and analysis, decision to publish, or preparation of the manuscript."

If this statement is not correct you must amend it as needed.

Please include this amended Role of Funder statement in your cover letter; we will change the online submission form on your behalf.

**Response**: Thank you for asking about the role of the funder. We have included the role of funder statement in the cover letter.

3. Thank you for stating the following in the Acknowledgments Section of your manuscript:

“We would like to acknowledge the HEPI initiative for providing funds to conduct this study.”

We note that you have provided funding information that is not currently declared in your Funding Statement. However, funding information should not appear in the Acknowledgments section or other areas of your manuscript. We will only publish funding information present in the Funding Statement section of the online submission form.

Please remove any funding-related text from the manuscript and let us know how you would like to update your Funding Statement. Currently, your Funding Statement reads as follows:

“This research was supported by a Health Professionals Education Partnership Initiative (HEPI) grant (grant number: R25TW011214) obtained from the US National Institutes of Health, Fogarty International Center.”

Please include your amended statements within your cover letter; we will change the online submission form on your behalf.

**Response**: We removed any funding-related text from the revised manuscript. We included the funding statement and role of funder statement in the cover letter.

4. Please include captions for your Supporting Information files at the end of your manuscript, and update any in-text citations to match accordingly.

**Response**: Thank you. We included a caption of the supplementary files at the end of the revised manuscript.

**Edits requested on the submission**

1. Thank you for uploading your study's underlying data set in the Supporting Information file "dataset.xlsx". We noticed that this file may contain potentially identifying participant information.

Before we proceed, we kindly ask that you please ensure that the data shared are in accordance with participant consent and all applicable local laws. If any data shared is not in accordance with participant consent, please remove these data and re-upload a fully anonymized data set. Please also note that spreadsheet columns with identifying information must be removed and not hidden as all hidden columns will appear in the published file.

**Response**: Thank you for the suggestion. The shared dataset does not contain any potentially identifying participant information such as names, addresses, images, or other unique identifiers. The dataset is fully anonymized. Additionally, the dataset aligns with participants’ consent agreements.

**Additional Editor Comments:**

I critically reviewed your article entitled “The hidden burden of hypertension-undiagnosed hypertension and associated factors among elderly individuals in Gedeo zone: A mixed methods approach” which has the potential to add to the existing body of scientific knowledge, particularly in developing countries. However, there are some limitations in your article that need addressing before publication.

**Response**: Dear editor, Thank you for giving us your valuable time and for sharing your valuable input.

1. There are several grammatical and typological errors that authors need to carefully review.

**Response**: Thank you. The revised manuscript has been meticulously edited and proofread to rectify quality issues, such as misspellings, grammatical errors, and unclear sentences.

2. Authors should extensively format manuscripts based on PLOS ONE journal style, including file naming. Avoid unnecessary italicizing and capitalization throughout the manuscript.

**Response**: Thank you for your suggestion. We prepared the revised manuscript according to the PLOS ONE requirements

3. Make sure that your reference contains all the necessary details and PLOS ONE style.

**Response**: Thank you for your suggestion. We have made sure the references fulfill the PLOS ONE guideline in the revised manuscript.

**Response to reviewer’s comments**

**Comments from Reviewer 1**

1. Thank you the authors working on the so called silent killer of undiagnosed hypertension.

**Response**: Thank you for giving us your valuable time to review our paper and for your comments and suggestions, which we got as valuable input to improve the manuscript.

2. This is a prospective study so that what was the difficulty of getting consent from each indiviadual or what does it mean for publication consent is not applicable?....

**Response**: Thank you. We did not face any difficulty obtaining consent, we got written consent from our participants as we explained in the ethical consideration section. We mentioned publication consent is not applicable because there was no personal identifier data or imaging that required publication consent.

3. exclusion criteria?,operational definition?

**Response**: Thank you for pointing this out. The inclusion and exclusion criteria were incorporated in the population section of the methods. Highlighted in Page 6, Line 113-116. We already included operational definition as “measurements” Page 8-10, Line 146-185.

4. some editting of language/expert involvement is better to be involved

**Response**: Thank you. We have edited the revised manuscript to rectify misspellings, grammatical errors, and unclear sentences.

**Comments from Reviewer 2**

1. The study is good for the body of knowledge and abstract and introduction was written in a clear , concise and intelligible manner.

**Response**: Thank you for giving us your valuable time to review our paper and for your comments and suggestions, which we got as valuable input to improve the manuscript.

2. The title should be modified to be clear

**Response**: Thank you for the suggestion. We modified the title to “Undiagnosed hypertension and associated factors among older adults in Gedeo zone, southern Ethiopia: A mixed methods approach”.

3. what is your background to say elderly 50 years or above? there is other age classification for elderly population

**Response**: Thank you for the insightful inquiry. Including hypertension, chronic diseases are more common among older adults (adults aged 50 and above). As proved by previous studies, the incidence of hypertension and other chronic illnesses increases as individuals age. Additionally, many studies in Africa used the age of 50 and above to define older adults. We have clearly explained the rationale for using these populations in the introduction section of the revised manuscript, page 5, lines 83-90. This study aims to measure undiagnosed hypertension among older adults who have been overlooked in previous studies. To ensure consistency, the term "elderly population" in the title was modified to "older adults".

4. Does the the district (3) selected for the study is representative for the Gedeo zone (13)?

**Response**: Thank you. As per the information we obtained from Gedeo zone administration, all the mentioned districts and town administrations belong to the Gedeo zone, which makes them representative of the zone.

5. Data management and analysis should be concise, clear and precise Eg. VIF and multicollinearity.

**Response**: Thank you. We rewrote the multicollinearity and VIF more clearly in the revised manuscript. Page 11-12, Lines 226-229.

6. For qualitative study what types of analysis method used? what does it mean thematic content analysis, it vague and not clear, please specify it. it creates mislead to qualitative result interpretation. in addition the qualitative result interpretation should be consistent/matched with analysis method.

**Response**: Thank you for the insightful inquiry. We would like to clarify that we employed thematic analysis for our qualitative study. The reference to thematic content analysis was made in error, and we have since corrected it to accurately reflect our use of thematic analysis. Regarding the consistency of our results with the method of analysis, we have revisited our interpretations and cross-verified them against our thematic analysis results. Each interpretation has been linked to specific themes in the revised manuscript, ensuring that the conclusions are grounded in the evidence presented.

7. what the qualitative study result support? please put the qualitative result in appropriate place

**Response**: Thank you for the suggestion. The results of qualitative analysis were placed after the quantitative result, page 18-21, Lines 308-362.

8. Please check the study for grammar and plagiarized content?

**Response**: Thank you. We have edited the revised manuscript to rectify misspellings, grammatical errors, and unclear sentences.

9. Majority (63.4%) of the study participants were age between 50 to 59 years, this classified as middle age adult. does it can say elder?

**Response**: Thank you for the insightful inquiry. Adults aged 50 and above are indeed not elderly rather they are adults who are relatively older (older adults). As we responded to question number 3, we explained why 50+ adults are our population, in the introduction section of the revised manuscript, page 5, lines 83-90.

10. What is the sample size for second objective?

**Response**: Thank you for your question. We have included the sample size calculation for both objectives in supplementary file 1 of the revised manuscript. We found that the sample size calculated for the second objective was lower, so we used the larger sample size from the first objective.

11. undiagnosed HTN is new so it is possible to say prevalence of undiagnosed HTN?

**Response**: Thank you for the insightful inquiry. We collect the data cross-sectionally, where the dependent and independent variables were collected simultaneously. Therefore, we could not determine whether the undiagnosed HTN was new or not. In order to calculate the incidence we were supposed to follow a group of people who were initially free from the undiagnosed HTN.

12. Why did you merge overweight and obesity? The degree of being obese and being overweight is different and could have different impacts.

**Response**: Thank you for the insightful inquiry. We merged being obese and overweight for the health and statistical context. In the health research context, both overweight and obesity are linked to increased risk of diseases, more specifically chronic diseases, though the degree of risk varies. This provides valuable insights for effective decision-making. Regarding the statistical perspective, merging variables leads to meeting the chi-square test criteria and improved statistical power to make comparisons.

13. Study period: replace the phrase “between March 19 and May 20, 2023” with “from March 19 to May 20, 2023”

**Response**: Thank you for the insightful suggestion. As per your suggestion, we modified the study period to ‘from March 19 to May 20, 2023’ in the abstract and methods section of the revised manuscript.

14. Regarding measurement of the dependent variable, two measurements were taken 5 minutes apart. I don’t think 5 minutes are enough to diagnose a patient with hypertension. Whenever possible, the diagnosis should not be made on a single office visit.

**Response**: Thank you for the insightful inquiry. We made the diagnosis in a single office visit to save time and avoid missing participants during follow-up visits to measure blood pressure. The repeated measurements were separated by 5 minutes. For proper measurement of blood pressure in an office in a sitting position, repeated measurements can be separated by 1-2 min ([10.1161/HYP.0000000000000087](https://doi.org/10.1161%2FHYP.0000000000000087)). Therefore, a 5-minute interval was quite enough.

15. Before measuring the blood pressure, have you addressed the protocols to measure blood pressure? For example, how do you assesses the participants to avoid smoking, caffeine and exercise for 30 min; empty bladder; remain seated and relaxed for 3–5 min? Nothing is written in this case as it could have impact on the result. Additionally, was the room Quiet with comfortable temperature?

**Response**: Thank you for pointing this out. We completely agree with you. We addressed all protocols to measure blood pressure before taking the blood pressure measurement. We waited for 30 minutes, after making sure that the participants were refrained from smoking cigarettes, drinking alcohol, or taking caffeine before their BP measurement was taken. This by default made them eligible for BP measurement as we confirmed they had not taken anything within 30 minutes. The participants were also told to empty their bladder and avoid drinking water shortly before the measurement. After they entered the measurement room, the participants sat in a chair with feet flat on the floor and back supported for 3 minutes before taking the first BP reading. The second measurements were taken 5 minutes later. We included this procedure in the revised manuscript. Page 8, lines 147-154

16. Regular physical activity was defined as Moderate intensity aerobic exercise (walking, jogging, cycling, yoga, or swimming) for 30 minutes on 5–7 days per week but in your case it is “Engaging in physical activity for 20–30 minutes, three days a week, is considered regular physical exercise”

**Response**: Thank you for the insightful inquiry. We measured physical activity according to the International Physical Activity Questionnaire (IPAQ), which is a widely used tool designed to assess physical activity levels in various populations globally. As per the IPAQ, a minimum of at least three activities in a week for 20-30 minutes per day is needed to be considered as a physical activity.

**Comments from Reviewer 3**

Although the manuscript is good, I have several criticisms. thus you'll attempt to review and edit in light of the feedback. I made to attach all comments in PDF format. concept, data analysis, and following the rules when writing the manuscript are some of those.

**Response**: Thank you for giving us your valuable time to review our paper and for your comments and suggestions, which we got as valuable input to improve the manuscript.

1. From the title line 1, there is a reputation for “hypertension-undiagnosed hypertension”, please rewrite again to get more credit or approval?

**Response**: Thank you for the suggestion. We modified the title to “Undiagnosed hypertension and associated factors among older adults in Gedeo zone, southern Ethiopia: A mixed methods approach”.

2. The abstract section line [13-14] points out that the only reason for the failure of the health system is that the cause of hypertension is not detected, please see other published documents ( <https://doi.org/10.1186/s12872-022-02733-3>)

**Response**: Thank you. In the first sentence of the background of the abstract, we tried to highlight that having an underdeveloped healthcare system is among the reasons most hypertension cases remained undiagnosed. As per your suggestion we modified the first sentence of the background of the abstract. Page 2, Line 13-14

3. Why are you selecting the elderly, please give real evidence of the selection of the elderly compared to other elderly?

**Response**: Thank you for the insightful inquiry. Chronic diseases, including hypertension, disproportionately affect older adults (adults aged 50 and above). Current health behaviors and the accumulated impact of harmful habits increase the likelihood of contracting chronic conditions in older age. In Ethiopia, there is a lack of research on undiagnosed hypertension in older adults. We incorporated the justification on page 5, lines 83-90.

4. Why choose only the Gedeo zone, southern Ethiopia, why not include other zones under southern Ethiopia?

**Response**: Thank you for pointing this out. There are studies conducted in Hawassa City, Wolayita sodo, and Gurage zone, however, there are no studies on undiagnosed hypertension in the Gedeo zone. Additionally, our study covers not just the urban area of Dilla town but also the rural areas in the zone.

5. In the section 19 of the method carried out among 609 randomly selected elderly people in the Gedeo area between 19 March and 20 May 2023, is this sample sufficient? Please analyze to add additional samples to get a more accurate result?

**Response**: Thank you for your suggestion. We have included the sample size calculation for both objectives in supplementary file 1 (S1 file) of the revised manuscript. We found that the sample size calculated for the second objective was lower, so we used the larger sample size from the first objective.

6. From line 27 of page 2 “The determinants that had a significant relationship were living in urban areas,” what about rural areas under the Gedeo region of southern Ethiopia?, because your study focused on the Gedeo region of southern Ethiopia, so you should also include rural populations.

**Response**: Thank you for your comment. We included both the urban and rural residents since the data was collected from city administrations and districts. We also used a variable ‘residence’ which was categorized as urban and rural. We found being an urban resident decreases the likelihood of undiagnosed hypertension as compared to being a rural resident. That is what we included in the abstract section.

7. In the result part line 26 pages 2 the last sentence is [33-34] is not appropriate position is better to write in the conclusion part.

**Response**: Thank you for your suggestion. We moved the last sentence of the result part of the abstract to the conclusion section in the revised manuscript.

8. From page 3 of the introduction paragraph 46, first sentences, "Non-communicable diseases (NCDs) are widely recognized as major obstacles to socio-economic progress worldwide," please give evidence or references to show its effect.

**Response**: Thank you. As per your suggestion, we cited the above sentence.

9. From the introduction part line [50-56] is about adults, but your investigation of older people, so the two ideas contradict, so please try to write a detailed introduction about older people? I do not think it is important to consider adult causes.

**Response**: Thank you for the insightful inquiry. As per your suggestion, we replaced the data about adults with older adults in the revised manuscript. Page 3, lines 49-55.

10. From line [81-82], “Despite the fact that undiagnosed hypertension is common in older people, to our knowledge, no research has been conducted in Ethiopia [19]’’, from this part there is no need for reference, because the sentence indicates your idea.

**Response**: Thank you for the suggestion. We removed the reference in the revised manuscript.

11. From line 6 [110-11], the phrase says: “As there has been no previous research on the prevalence and associated factors of undiagnosed hypertension among elderly people in Ethiopia.”, your study area focused only on a restricted Gedeo area, southern Ethiopia, but there are so many studies done in Ethiopia, Africa and other countries, so how unique is your research from other research?

**Response**: Thank you for the insightful inquiry. Indeed there are a number of studies in Ethiopia on the prevalence and determinants of undiagnosed hypertension. However, these studies were conducted among the adult population. Our study added value by incorporating only older adults since the disease is more prevalent among the older population.

12. From line 6[117-119] of page 6 the sentence says: "Then a total of 14 kebeles were chosen from each designated county by the lottery method to obtain 30% of the kebeles in that county", is that a standardized accepted method for the use of the lottery method?

**Response**: Thank you. As a rule of thumb, it is recommended to use one-third in the lottery method to increase the representativeness of the data. Since it was not affordable by time and cost to include all population in the study.

13. On page 11 of line 216, the phrase states "For qualitative data, all interviews recorded have been verbatim translated into the local Amharic language", but your research area Gedeo zone, southern Ethiopia, why choose Amharic language?, because Gedeo zone, southern Ethiopia has its own language.

**Response**: Thank you. Those who speak Amharic language were interviewed in Amharic and those who do not speak it (most of the rural participants) were interviewed in Gedeo’ffa language. We modified the phrase in the revised manuscript. Page 12, lines 230-231.

14. From page 22 in line 414, the sentence says, "Another disadvantage may be the participant's reporting of prejudices," how do you know it and how to solve it? Because reporting biases occur, there is a question about data quality.

**Response**: Thank you for the insightful inquiry. When we say there might be a reporting bias, this means participants might have a social desirability bias in which their responses align with expected responses. However, there was no discrepancy in reports which might indicate the presence of social desirability bias.

15. From the reference, please use the most recently published document not the oldest published document? For example, in 2012 more than a decade, so it is not a relevant reference.

**Response**: Thank you for your suggestion. We utilized recent references in the revised manuscript. However, for certain sources, such as guidelines, we included older references because they represent the original guidelines from which subsequent citations were derived.
