# Supplementary material for: Undiagnosed hypertension and associated factors among older adults in Gedeo zone, southern Ethiopia: A mixed methods approach
Source: PLoS One. 2025 May 5;20(5):e0322610. doi: 10.1371/journal.pone.0322610 (PMC12052162; doi:10.1371/journal.pone.0322610)
Supplement: Attachment — Submitted filename: Response_to_reviewers_auresp_2.docx [file pone.0322610.s007.docx]

**Subject: Responses to comments (revision 2)**

Dear Editor,

Thank you for taking the time to consider our manuscript titled **“**Undiagnosed hypertension and associated factors among older adults in Gedeo zone, southern Ethiopia: A mixed methods approach**”** for the Plos One Journal original research article. We appreciate the time and effort you and the reviewers have dedicated to providing valuable feedback on our manuscript.

We have considered the comments and concerns and made every effort to address them. We agree with all the comments and have incorporated the corresponding revisions into the revised manuscript. All revised texts are track-changed to point out the changes we made. We believe that our manuscript has been significantly improved as a result of these revisions.

We would like to thank you once again for your consideration of our work and for inviting us to submit the revised manuscript. We look forward to hearing from you. Our detailed, point-by-point responses to the comments are given below.

Best regards,

Tsion Mulat Tebeje

School of Public Health, Dilla University, Dilla, Ethiopia.

Email: [yemarina12@gmail.com](mailto:yemarina12@gmail.com) / [Tsionmulat@du.edu.et](mailto:Tsionmulat@du.edu.et) (corresponding author)

Journal Requirements

1. Please review your reference list to ensure that it is complete and correct. If you have cited papers that have been retracted, please include the rationale for doing so in the manuscript text, or remove these references and replace them with relevant current references. Any changes to the reference list should be mentioned in the rebuttal letter that accompanies your revised manuscript. If you need to cite a retracted article, indicate the article’s retracted status in the References list and also include a citation and full reference for the retraction notice.

**Response**: We have reviewed our list of references and changed some references. We have removed and replaced the retracted articles with relevant and current references. All changes made to the reference list have been highlighted in the revised manuscript using track change.

Response to reviewers

Reviewer 2

**General comment**: Thank you very much to get the opportunity to review this important public health problem. The authors are fully addressed the comment I raised previously. but I have some suggestions to improve quality and scientific back ground of the manuscript.

**Response:** Thank you for your kind words and for reviewing our manuscript which benefited from your comments and suggestions.

**Comment 1**: What adds this study from qualitative finding?

**Response 1**: The qualitative study aimed to assess participants' knowledge and attitudes towards hypertension. Participants' knowledge is one aspect measured in the quantitative portion of this research. Consequently, the qualitative findings provided further insight into their understanding of hypertension, including its definition, signs and symptoms, self-management strategies, and preventive measures. This approach revealed additional concepts regarding knowledge about hypertension that are not addressed in the quantitative questions.

**Comment 2**: In discussion parts the qualitative study have not support the quantitative finding simply put in separate manner and compares with other similar study only. what is the reason to add qualitative study to this research? I don.t see its relevance as it stands.

**Response 2**: Thank you. As we mentioned above the qualitative study is meant to assess participants' knowledge and attitudes towards hypertension. We aligned the qualitative and quantitative study in the discussion section.

**Comment 3**: the objective is the same for both quantitative and qualitative, but it seems like different in your write up. it needs arrangement. for example you should explained the quantitative study, then followed by qualitative finding to make the finding strong.

**Response 3**: Thank you for your insightful suggestion. In the results section, we started with quantitative results, followed by qualitative findings.

**Comment 4**: non of the participants knew the scientific back ground of the HTN. It needs modification.

**Response 4**: Thank you. We have changed it to “few of them knew the scientific definition of HTN” and we made the changes with track change to spot the changes.

**Comment 5**: The qualitative analysis in some sentences seems like to summative content analysis. it needs some modification.

**Response 5**: Thank you. As per your suggestion, we have modified the parts that may seem summative content analysis. Page, lines

**Comment 6**: quantitative and qualitative study lack integrity at discussion parts of the manuscript. it needs align together.

**Response 6**: Thank you for pointing this out. In the discussion section, we integrated the qualitative findings with the quantitative findings.
